# The History and Capabilities of Herbal Simples

**Published:** 1890-07-12

**Authors:** W. T. Fernie


### The History and Capabilities of Herbal Simples.

By W. T. Fernie, M.D.

(*Continued from page* 196.)

### XIV.—CARAWAY—CARROT.

### Caraway.

The common Caraway—Carum Carui—might almost say with George Canning's Needy Knifegrinder, "Story ! God bless you ! I have none to tell, Sir !" It is a plant of the umbelliferous order, and hailed originally from Caria, in Asia Minor, whence has come its name. But it has long been naturalised in England, and was known here in Shakespeare's time, who makes mention of it in the second part of Henry IV., thus, "Come, Cousin, silence; we will eat a pippin of last year's graffing, with a dish of caraways; and then to bed." It is freely cultivated in Kent and Essex. The seeds, which grow numerously in the small flat flowers placed thickly together on each umbel, furnish, when bruised, a spicy smell, and a warm aromatic taste. They are cordial, and stomachic, giving relief in flatulency and hysteria. We know them best in seed cake, and as caraway comfits. But why the wife of a comfit-maker should be given to swearing, as Shakespeare avers, it is not easy to see. These comfits consist of the seed encrusted with white sugar. The young roots of caraway plants may be eaten at table like parsnips; they warm and comfort a cold, weak stomach. These mixed with milk, and made into bread, formed the *chara* of Julius Cæsar, eaten by the soldiers of Valerius. Chemically, the volatile oil obtained from the seeds consists of carvol, and a hydro-carbon, carvene, which is a sort of camphor. From two to four drops of this oil may be taken on sugar, as a carminative dose. Dioscorides recommended it long ago for pale-faced girls; and modern ladies have not disregarded this advice. From six pounds of the unbruised seeds four ounces of the pure essential oil can be obtained. In Germany the peasants flavour their cheese, soups, and household bread with the caraway. This is not a modern custom; for an old Latin author says: "Semina carui satis communiter adhibentur ad condiendum panem; et rustici nostrates esitant jusculum e pane seminibus carui, et cerevisia coctum." In our own rural districts country persons pound a hot loaf fresh from the oven, with a handful or more of the seeds, and wetting the whole with brandy or whiskey, apply it over the part for deep seated ear-ache or for face-ache. An ounce of the bruised seeds infused for six hours in a pint of cold water makes a good caraway water for infants, from one to three teaspoonfuls for a dose. It "consumeth winde, and is delightful to the stomack. The powdered seed put into a poultis taketh away blacke and blew spots of blowes and bruises." The oil or seeds of caraway sharpen the vision, and promote the secretion of milk. Short-sighted men and nursing mothers may therefore courageously indulge in seed cake. As regards its stock of honey the caraway may be termed in a double sense, like Uriah Heep, "truly umbel." The diminutive florets clustered together on its flat disk are so shallow that Lepidopterous and Hymenopterous insects, with their long probosces, stand no chance of getting a meal. They fare as ill as the stork did in the fable, whom the fox invited to dinner served on a soup plate. It is only the smaller fry of insects with the mouths of babes and sucklings, which can take a seat at this modest board. Accordingly, as Sir John Lubbock has shown, out of fifty-five visitants to the caraway for nectar, one moth, nine bees, twenty-one flies, and twenty-four miscellaneous midges constituted the dinner party.

### The Carrot.

Our garden carrot is a cultivated variety of the Daucus sylvestris, or wild carrot, "which groweth of itself in untoiled places, and is called philtron, because it serveth for love matters." The garden carrot was first introduced in the reign of Elizabeth, and was then so highly esteemed that the ladies wore leaves of it in their head dresses. But as long ago as in the time of Edward III. an edible carrot had been developed from the wild plant by the skill of the Flemings. This wild carrot is common in pasture grounds, and fallow fields, and road sides. It belongs to the umbelliferous order, and is also called bird's nest, because the stems of its flowering head or umbel, form a concave circle or nest, which bees, when belated from the hive, love to use as a dormitory. The small purple flower which grows in the middle of the umbel has been found useful for the cure of epilepsy. The juice of the carrot contains carotine, in red crystals, also pectine, albumen, and a particular volatile oil, on which the medicinal properties of the root depend. The seeds are warm, and aromatic to the taste; whilst they are slightly diuretic. A tea made from the whole herb, and taken each night and morning is excellent when the lithic acid or gouty disposition prevails with a reddish sediment in the urine, on its becoming cool. Carrots, if boiled long enough, and mashed into a pulp, when applied directly to a putrid, indolent sore, will sweeten and heal it. The carrot poultice was first discovered by Sulzer; it mitigates pain, and corrects the stench of foul ulcers. Raw scraped carrot is an excellent application for chapped nipples. The peasants of Savoy use an infusion of carrots as a specific for jaundice. Before the French Revolution the sale of carrots and oranges was prohibited in the Dutch markets, because of the unpopular aristocratic colour of these commodities. As a vegetable at table the carrot is not very easy digestible. The red part is more valuable as an esculent than the yellow portion of the root. In one thousand parts of a carrot are ninety-five of sugar, and three of starch. Its name is due either to the Latin caro—flesh, as a fleshy vegetable; or to the Celtic, car—red, because of its colour. In country districts raw carrots are given to children for expelling worms, probably because the vegetable matter passes through the body without undergoing much change. "Remember, William," says Sir Hugh Evans, in the Merry Wives of Windsor, "Focative is Caret." "And that," replies Mrs. Quickly, "is a good root."

